# Combined Quantification of the Global Proteome, Phosphoproteome, and Proteolytic Cleavage to Characterize Altered Platelet Functions in the Human Scott Syndrome*[Fn FN2]

**DOI:** 10.1074/mcp.M116.060368

**Published:** 2016-08-17

**Authors:** Fiorella A. Solari, Nadine J.A. Mattheij, Julia M. Burkhart, Frauke Swieringa, Peter W. Collins, Judith M.E.M. Cosemans, Albert Sickmann, Johan W.M. Heemskerk, René P. Zahedi

**Affiliations:** From the ‡Leibniz-Institut für Analytische Wissenschaften-ISAS-e.V., Dortmund, Germany;; §Department of Biochemistry, Cardiovascular Research Institute Maastricht (CARIM), Maastricht University, Maastricht, The Netherlands;; ¶Arthur Bloom Haemophilia Centre, School of Medicine, Cardiff University, Cardiff, United Kingdom;; ‖Medizinisches Proteom-Center, Ruhr-University Bochum, Bochum, Germany;; **Department of Chemistry, College of Physical Sciences, University of Aberdeen, Aberdeen, UK

## Abstract

The Scott syndrome is a very rare and likely underdiagnosed bleeding disorder associated with mutations in the gene encoding anoctamin-6. Platelets from Scott patients are impaired in various Ca^2+^-dependent responses, including phosphatidylserine exposure, integrin closure, intracellular protein cleavage, and cytoskeleton-dependent morphological changes. Given the central role of anoctamin-6 in the platelet procoagulant response, we used quantitative proteomics to understand the underlying molecular mechanisms and the complex phenotypic changes in Scott platelets compared with control platelets. Therefore, we applied an iTRAQ-based multi-pronged strategy to quantify changes in (1) the global proteome, (2) the phosphoproteome, and (3) proteolytic events between resting and stimulated Scott and control platelets. Our data indicate a limited number of proteins with decreased (70) or increased (64) expression in Scott platelets, among those we confirmed the absence of anoctamin-6 and the strong up-regulation of aquaporin-1 by parallel reaction monitoring. The quantification of 1566 phosphopeptides revealed major differences between Scott and control platelets after stimulation with thrombin/convulxin or ionomycin. In Scott platelets, phosphorylation levels of proteins regulating cytoskeletal or signaling events were increased. Finally, we quantified 1596 N-terminal peptides in activated Scott and control platelets, 180 of which we identified as calpain-regulated, whereas a distinct set of 23 neo-N termini was caspase-regulated. In Scott platelets, calpain-induced cleavage of cytoskeleton-linked and signaling proteins was downregulated, in accordance with an increased phosphorylation state. Thus, multipronged proteomic profiling of Scott platelets provides detailed insight into their protection against detrimental Ca^2+^-dependent changes that are normally associated with phosphatidylserine exposure.

The Scott syndrome is a very rare, moderately mild bleeding disorder, clinically identified by a reduced prothrombin consumption of the blood serum. Platelets and other blood cells from Scott patients show a deficiency in Ca^2+^-dependent membrane phospholipid scrambling (1, 2). As a result, Scott platelets are greatly impaired in externalization of the aminophospholipids, phosphatidylserine (PS)^1^, and phosphatidylethanolamine in response to strong, Ca^2+^-mobilizing agents like collagen/thrombin or ionomycin (3–6). This leads to a severe reduction in the binding of several coagulation factors in Scott syndrome platelets, which explains the bleeding phenotype in these rare patients (7). So far, one family and two independent patients with Scott syndrome have been described in the literature. In addition, a breeding of dogs with impaired hemostasis is known with the same platelet phenotype (8). On the other hand, the Scott syndrome as a moderately mild bleeding disorder likely is underdiagnosed, because the indicative lab diagnostics (prothrombin consumption test or PS exposure) are not regularly performed.

In 2010, a critical role was reported for the transmembrane protein anoctamin-6 (gene *ANO6,* alias *TMEM16F*), in the Ca^2+^-dependent PS exposure of platelets (9). A role of anoctamin-6 in Scott syndrome was postulated by the discovery of dysfunctional mutations in the *ANO6* gene of two unrelated patients (9, 10). This anoctamin is also known as a Ca^2+^-dependent ion channel with permeability to both chloride ions and cations (10), which activity appeared to be defective in Scott cells (5, 9). A dysfunctional mutation in *ANO6* (*TMEM16F*) has also been identified in a breeding of dogs with impaired hemostasis (8).

Recent findings however question if a deficiency of anoctamin-6 alone can account for the complex phenotype of Scott syndrome platelets. The platelets from a patient with *ANO6* mutations and from anoctamin-6 deficient mice show a complex phenotype: along with agonist-induced PS exposure, closure of activated integrins appears to be affected, as well as calpain-dependent cleavage of intracellular proteins and cytoskeletal-dependent swelling of the platelets (6, 11, 12). Given this, we expected that extended proteomics analysis will provide important novel information on the possible roles of other proteins than anoctamin-6 in the altered properties of Scott platelets.

For the present work, we hypothesized that the complex phenotypical changes in Scott platelets are a consequence of multiple alterations in the platelet signaling machinery, directly or indirectly related to the absent anoctamin-6 expression, and that these alterations may provide insights into the mechanisms underlying the important procoagulant response. Thus, we compared platelets isolated from healthy controls and a diagnosed Scott patient in terms of functional (procoagulant) activity in relation to changes in protein levels, phosphorylation patterns, and proteolytic cleavage. Control and Scott platelets were therefore activated with (1) thrombin, (2) convulxin/thrombin, and (3) ionomycin, representing different levels of procoagulant activity. Given major differences in protein expression levels between human and mouse platelets, we confined this work to the human system.

For the complex proteome analysis, we applied iTRAQ stable isotope labeling in conjunction with TiO_2_ phosphopeptide enrichment and high pH reversed phase fractionation, allowing simultaneous quantitative analysis of the global proteome and phosphoproteome of (activated) Scott and control platelets (figure 1). Such mass spectrometry-based techniques have previously been used for a separate quantification of the majority of proteins (13, 14) and regulated protein phosphorylation sites in platelets isolated from healthy subjects (15, 16). We also applied our recently developed charge-based fractional diagonal chromatography (ChaFRADIC) approach to identify neo-N-terminal peptides, produced upon proteolytic activity (17–20). This allowed us to distinguish between calpain- and caspase-mediated protein cleavage patterns in Scott and control platelets. To our knowledge, this is the first time that such a broad combination of proteomics technologies has been used to assess the (post-translational) protein changes in a human blood cell, here platelets, isolated from, to our knowledge, the only available Scott patient worldwide.

## EXPERIMENTAL PROCEDURES

### 

#### Materials

Convulxin was purified to homogeneity from crude snake venom (21). Proteinase-activated receptor-1 (PAR-1) agonist peptide SFLLRN was from Bachem (Bubendorf, Switzerland), human α-thrombin and ionomycin were from Sigma-Aldrich (St. Louis, MO). Fluorescein isothiocyanate (FITC)-labeled anti-CD62P mAb against P-selectin was from Beckman Coulter (Marseille, France); FITC-annexin A5 from PharmaTarget (Maastricht, The Netherlands); Alexa Fluor (AF)647-labeled fibrinogen from Invitrogen Life Technologies (Bleiswijk, The Netherlands); ABT-737 from Santa Cruz Biotechnology (Santa Cruz, CA); QVD-Oph and calpeptin from Calbiochem (San Diego, CA). Other chemicals, (ant)agonists and antibodies were obtained from sources described before (11).

##### Patient and Control Subjects

Blood was obtained from four healthy volunteers and a diagnosed patient with Scott syndrome (Scott_UK_, the only one available worldwide for blood donations) after full informed consent (Helsinki declaration). The Scott_UK_ patient has been characterized as heterozygous for two mutations in the *ANO6* alleles: a transition at the first nucleotide of intron 6 (IVS6 + 1G→A), disrupting the splice site consensus sequence of intron 6, and a single-nucleotide insertion in exon 11 (c.1219insT), predicting a frameshift at codon 411 (10). Both mutations are not compatible with normal translation. Not available to this study were the two other described Scott patients (1, 2). However, mutations in *ANO6* have also been identified in these patients from the United States (9) and France (F. Toti, personal communication). Protocols were approved by the local Medical Ethics Committees.

##### Blood Collection and Platelet Isolation

Blood samples were collected into 1/6 volume of acid-citrate glucose solution (ACD, 80 mm trisodium citrate, 52 mm citric acid, and 180 mm glucose). Platelet-rich plasma (PRP) was obtained by centrifuging at 260 × *g* for 15 min (11). After addition of 1/15 volume of ACD, platelets were pelleted by centrifugation at 870 × *g* for 15 min. To obtain highly purified platelets, pellets were resuspended in Hepes buffer pH 6.6 (10 mm Hepes, 136 mm NaCl, 2.7 mm KCl, 2 mm MgCl_2_, and 0.1% glucose) by carefully excluding the bottom layer of red blood cells. After addition of ACD (1/15 volume) and apyrase (1 U/ml), the cells were centrifuged again at 2000 × *g* for 5 min in an Eppendorf centrifuge, and resuspended in Hepes buffer pH 7.45 (10 mm Hepes, 136 mm NaCl, 2.7 mm KCl, 2 mm MgCl_2_, 0.1% glucose), again by excluding the bottom layer of erythrocytes. Purity of the final platelet suspensions was checked with a Thrombocounter and from microscopic preparations. Contamination with red blood cells was <1:15,000, contamination with leukocytes was <1:20,000. Blood samples were used for measurements of platelet phenotype and for platelet proteomic analysis in parallel.

##### Platelet Stimulation and Thrombus Formation

Purified, washed platelets (controls and Scott patient) in Hepes buffer pH 7.45 (10 mm Hepes, 136 mm NaCl, 2.7 mm KCl, 2 mm MgCl_2_, 0.1% glucose) containing 2 mm CaCl_2_ were left untreated or were activated with thrombin, thrombin/convulxin, or ionomycin for 30 min at 37 °C under nonstirring conditions. This time point was chosen, based on earlier findings that 30 min of activation with agonists was required for near maximal PS exposure and integrin closure/cleavage in control platelets (11, 12).

For obtaining reference values for apoptosis-induced caspase protein substrates, platelets were treated for 1 h with the BH3 mimetic ABT-737, an agent that is in study for the therapeutic targeting of the Bcl-2 family of prosurvival proteins in antitumor treatment. This compound provides a standard way to induce apoptotic PS exposure in platelets (6, 22).

For proteomics (global, phospho, N termini) analysis, platelet samples (5.0 × 10^8^/ml) were collected into 1 volume of lysis buffer (50 mm Tris, 1% SDS, 150 mm NaCl, 1 tablet PhosStop/7 ml, pH 7.8) (15). Lysed samples were immediately frozen and stored at −80 °C until usage. Parallel samples (1.5 × 10^8^/ml) were analyzed by flow cytometry for PS exposure using FITC-labeled annexin A5, as described before (6).

Collagen-induced thrombus formation was assayed using PPACK/fragmin-anticoagulated human blood (controls and Scott patient), as described (12). Blood samples were perfused over a collagen surface at shear rate of 1000/s for 4 min. Thrombi formed were poststained with AF647-annexin A5 and FITC-anti-CD62P mAb. Phase-contrast and fluorescence images were recorded to determine platelet deposition, P-selectin expression, and PS exposure (12).

##### Western Blot Analysis

Washed platelets (5 × 10^8^/ml) were left untreated or activated by convulxin (200 ng/ml) plus thrombin (8 nm) or ionomycin (20 μm) in the presence of 2 mm CaCl_2_ for 30 min at 37 °C under nonstirring conditions. Samples of resting and activated platelets were lysed with ice-cold 4× lysis buffer (600 mm NaCl, 10 mm Tris, 4 mm EGTA, 4 mm EDTA, 4% Nonidet P-40). Samples (10 μg proteins) were separated on 8% SDS-PAGE gels, and transferred to PVDF blotting membranes. Immunoblotting was with the following antibodies: Ab762 against the full length β_3_-chain (1:10000), Ab754 against the intracellular amino acid 754 neo-N terminus of the cleaved β_3_-chain (1:1000), or α-tubulin (1:1000) (12). Incubation with secondary horse radish peroxidase-coupled antibody (1:1000) was overnight at 4 °C. Blots stained with an ECL system were quantified by densitometric analysis.

##### Sample Digestion and Preparation for Quantitative (Phospho)Proteomics

Platelet proteomics analyses were performed, based on procedures reported earlier (13, 15, 23), with modifications. Well purified platelet samples (5 × 10^8^ platelets) were prepared in SDS lysis buffer, and protein concentrations were determined using a bicinchoninic acid assay (Pierce, Thermo-Fisher Scientific, Bremen, Germany). Cysteines were reduced by 30 min incubation at 56 °C with 10 mm dithiothreitol; free sulfhydryl groups were alkylated with 30 mm iodoacetamide for 30 min at room temperature. Samples of 150 μg protein were then processed using filter-aided sample preparation (FASP) with a 30 kDa molecular weight cut-off spin filter (24, 25).

Proteins were digested in 50 mm triethylammonium bicarbonate (TEAB), 200 mm guanidinium hydrochloride, and 2 mm CaCl_2_, pH 8.0 in the presence of trypsin (1:20 w/w, T-1426, Sigma, St. Louis, MO) at 37 °C. After incubation for 7 h, digested peptides were collected by centrifugation at 13,800 × *g* for 25 min. Filters were washed with 50 μl of 50 mm TEAB and 50 μl of LC-MS grade water to increase peptide yield. Trypsin digestion was monitored by monolithic RP HPLC as described (23). After lysis and digestion with trypsin, samples were used for either label-free proteome analysis, or iTRAQ-based (phospho)proteome analysis.

##### iTRAQ Labeling and Pooling

**Fig. 1.**
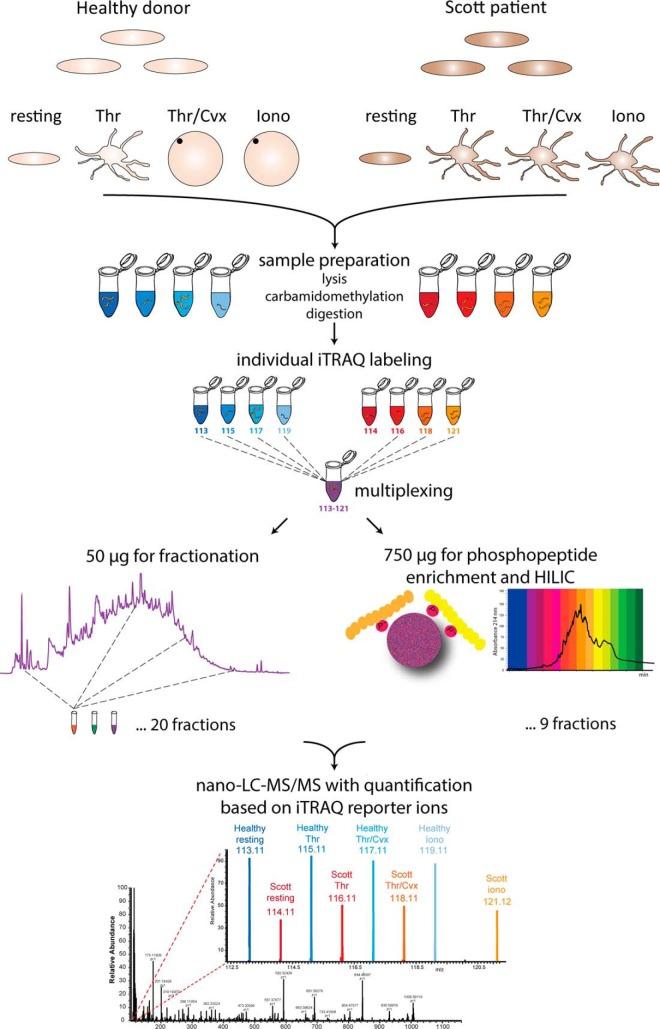
**Strategy and workflow for combined determination of platelet quantitative (phospho)proteomics.** Purified washed platelets (4 × 10^8^) from a healthy donor and the Scott patient in CaCl_2_-containing medium remained resting or were stimulated with thrombin (8 nm), thrombin/convulxin (200 ng/ml, 8 nm) or ionomycin (20 μm) for 30 min. After lysis and digestion with trypsin, all eight samples were individually labeled with 8-plex iTRAQ reagent (113, 114, 115, 116, 117, 118, 119, and 121). For assessment of the quantitative proteome and the phosphoproteome, the eight samples were pooled in a 1:1 ratio; 10% of the pooled sample was used for global proteome analysis and the remaining 90% for phosphoproteome analysis.

The workflow for assessment of the global platelet proteome and platelet phosphoproteome is schematized in Figure 1. Samples were dried under vacuum and reconstituted in iTRAQ 8-plex dissolution buffer (AB Sciex, Dreieich, Germany), followed by individual labeling with iTRAQ 8-plex labels (113, 114, 115, 116, 117, 118, 119, and 121) according to the manufacturer's protocol, and pooling. The pooled peptide sample was desalted by C_18_ solid phase extraction (SPEC C_18_ AR, 4 mg bed; Agilent Technologies, Brussels, Belgium), dried under vacuum, and ∼6% was reconstituted into 10 mm ammonium acetate pH 6.0 (solvent A) for high pH reversed phase (RP) fractionation and subsequent quantification of the global proteome. The remaining ∼94% of the sample was used for phoshopeptide enrichment (see below).

##### Platelet Global Proteome Analysis Using iTRAQ Labels

Fifty micrograms of the iTRAQ-labeled pooled peptide sample were separated by RP at pH 6.0. Therefore, the sample was loaded on a C_18_ column (BioBasic-18, 5 μm particle size; 300 Å pore size, 150 × 0.5 mm), using a binary gradient at flow rate of 12.5 μl/min ranging from 3–60% of 10 mm ammonium acetate and 84% acetonitrile, pH 6.0, in 70 min. A total of twenty concatenated fractions were collected, and analyzed by nano LC-MS/MS in data-dependent acquisition (DDA) mode, using a U3000 nano-RSLC system online-coupled to a Q-Exactive mass spectrometer. Half of each fraction was loaded onto a trap column (Acclaim PepMap100 C_18_; 100 μm × 2 cm) with 0.1% trifluoroacetic acid (TFA). Peptides were separated on the main column (PepMap100 C_18_; 75 μm × 50 cm), using a binary gradient ranging from 3–42% solvent B (84% acetonitrile, 0.1% formic acid) in 100 min at 60 °C and a flow rate of 270 nL/min. In the Q-Exactive, survey scans were acquired at resolution of 70,000, using the polysiloxane *m*/*z* 371.1012 as lock mass (26), with an automatic gain control target value of 3 × 10^6^. Subsequently, MS/MS spectra of the 15 most intense ions were acquired with (1) a resolution of 17,500, (2) an isolation width of 2.0 *m*/*z*; (3) a normalized collision energy of 35; (4) an AGC target value of 1 × 10^6^ ions; (5) a maximum injection time of 250 ms; (6) a dynamic exclusion of 12 s and an underfill ratio of 10%. The first fixed mass was set to 105 *m*/*z*. Reaction tubes containing 10% ammonium water were placed in front of the ion source, in order to compensate for the iTRAQ-induced increase of peptide charge states (27).

Raw data were processed with Proteome Discoverer 1.4 (Thermo-Fisher Scientific) using the Uniprot human database (August 2012; 20,232 target sequences). Mascot and Sequest were applied as search algorithms with the following settings: (1) trypsin as enzyme allowing two missed cleavages, (2) iTRAQ 8-plex at N termini and lysine (+304.2053 Da) and carbamidomethylation of cysteine (+57.0214 Da) residues as fixed modifications, (3) oxidation of methionine (+15.9949 Da) as variable modification, (4) mass tolerances for MS and MS/MS were set to 10 ppm and 0.02 Da, respectively. False discovery rate (FDR) estimation on the level of peptide spectrum matches (PSM) was performed using the peptide validator node with filtering for 1% FDR (high confidence filter). The reporter ion quantifier node was used for iTRAQ reporter quantification. Only proteins quantified with at least two unique peptides were considered. Details for the data analysis are given in the supplemental methods.

##### Label-free Analysis of Global Platelet Proteome

In addition we conducted a label-free quantification workflow using the unstimulated Scott and healthy platelet samples. An advantage of this strategy is that it is not affected by potential co-isolation interference frequently observed in reporter ion-based quantification methods (28, 29). Therefore, 500 ng of untreated healthy and Scott platelets were analyzed by nano-LC-MS/MS in DDA mode, using a U3000 nano-RSLC system online-coupled to an Orbitrap Fusion mass spectrometer, with LC parameters as above. Survey scans were acquired at a resolution of 120,000, using the polysiloxane *m*/*z* 445.1200 as lock mass (26), an automatic gain control target value of 4 × 10^5^, and a maximum injection time of 50 ms. The top 20 precursor ions were selected for fragmentation by HCD with a collision energy of 27 and MS/MS were acquired in the Orbitrap using a target value of 5 × 10^4^ ions, a maximum injection time of 50 ms, and a dynamic exclusion of 30 s.

Data analysis for label-free quantification was performed using Progenesis LC-MS software version 4.1 from Nonlinear Dynamics (Newcastle upon Tyne, UK), in combination with PeptideShaker 1.9.2 (30). After alignment peak lists were exported and searched against a concatenated target/decoy version of the human Uniprot database (December 2013; 20,274 target sequences), using X! TANDEM Vengeance (version 2015.12.15.2), Mascot 2.4 (Matrix Science), MS-GF+ Beta (v10282) (12/19/2014), OMSSA and SearchGUI 2.8.1 (28). The following search parameters were used: (1) trypsin as protease with a maximum of two missed cleavages, (2) carbamidomethylation of Cys (+57.0214 Da) as fixed, and (3) oxidation of Met (+15.9949 Da) as variable modification. MS and MS/MS tolerances were set to 10 ppm and 0.02 Da. Search results were combined using PeptideShaker 0.29.1, filtered at an FDR of 1% and reimported into Progenesis. Peptide sequences containing oxidized Met and pyro-Glu obtained from the second pass X!Tandem search were omitted from further data analysis, and only proteins quantified with at least two unique peptides were considered for quantification. For all proteins, ratios between platelet samples were calculated based on normalized areas obtained from Progenesis. Next, after log 2 transformation mean and standard deviations were calculated and regulation factors were defined as described in the Experimental Design and Statistical Rationale section (± 2 × S.D. (2 × 0.535)).

##### Platelet Phosphoproteome Analysis

For phosphopeptide analysis of the pooled iTRAQ samples, a TiO_2_-based phosphopeptide enrichment protocol was conducted, as reported by Larsen and co-workers (31). Samples were dried under vacuum and resuspended in TiO_2_ loading buffer (80% acetonitrile, 5% TFA, and 1 m glycolic acid). TiO_2_ beads were added in a bead-to-peptide ratio of 6:1. The suspension was incubated for 10 min at RT, and centrifuged to pellet the beads containing the phosphopeptides. This step was repeated twice using 1/2 and 1/4 of the amount of beads. Beads were pooled and washed with 80% acetonitrile, 1% TFA, followed by a second washing step with 10% acetonitrile, 0.1% TFA. Phosphopeptides were eluted with 1% ammonium hydroxide, pH 11.3. The eluted phosphopeptides were recovered and dried under vacuum, followed by a second enrichment step using loading buffer 2 (70% acetonitrile, 2% TFA). The eluates were acidified using TFA (pH <2) and desalted using Oligo R3 microcolumns. Enriched phosphopeptides were fractionated with a U3000 nano-RSLC system in hydrophilic interaction liquid chromatography (HILIC) mode. Peptides were loaded on a self-packed HILIC column (Polar phase TSKgel Amide-80; 150 μm ID × 15 cm length; 5 μm particle size; 80 Å pore size, Tosoh Bioscience, Stuttgart, Germany), and separated using a binary gradient ranging from 5–40% solvent B (solvent A: 98% acetonitrile, 0.1% TFA; solvent B: 0.1% TFA) within 40 min at a flow rate of 4 μl/min. Eluting peptides were collected in nine fractions.

All HILIC fractions were analyzed by nano-LC-MS/MS in DDA mode using an U3000 nano system, online coupled to an Orbitrap Velos mass spectrometer (Thermo-Fisher Scientific). Peptides were preconcentrated on a self-packed precolumn (Kinetex; 2.6 μm particle size; 100 Å pore size) and further separated on a self-packed main column (Kinetex, 75 μm ID × 30 cm length; 2.6 μm particle size; 100 Å pore size) using a binary gradient as above, however ranging from 3–45% B in 75 min at a flow rate of 250 nL/min and 60 °C. Survey scans were acquired with a resolution of 30,000 using the polysiloxane *m*/*z* 371.1012 as lock mass (26). Subsequently, HCD MS/MS spectra of the five most intense ions were acquired in the Orbitrap with (1) a resolution of 7500 (2) an isolation width of 2.0 *m*/*z*; (3) a normalized collision energy of 50; (4) an AGC target value of 1 × 10^5^ ions; (5) a maximum injection time of 200 ms; (6) and a dynamic exclusion of 12 s. Reaction tubes containing 10% ammonium water were placed in front of the ion source (27).

##### Data Analysis for Quantitative Phosphoproteomics

Phosphoproteomic raw data were processed with Proteome Discoverer 1.3 (Thermo-Fisher Scientific), using Sequest and Mascot as search algorithms and the same parameters as used for the global iTRAQ proteome. Additionally, phosphorylation of Ser/Thr/Tyr (+79.9663 Da) was selected as a variable modification and probabilities for phosphorylation site localizations were determined using phosphoRS (32). PSMs were filtered for 1% FDR (high confidence filter), and only phosphopeptides with phospho-RS probabilities ≥90% were considered as confident.

iTRAQ ratios obtained from Proteome Discoverer were processed as described for the global iTRAQ data and normalized using the iTRAQ global proteome normalization factors in order to compensate for systematic errors (see supplemental Methods).

For determination of confident phosphorylation sites, a ready-to-use Excel macro provided by Mechtler lab (http://ms.imp.ac.at) was used. Peptides were grouped based on their sequences, protein accessions, and phosphorylation sites. Next, for each PSM, normalized abundance values were calculated as for the global proteome. For grouped PSMs representing the same protein, phosphopeptide sequence or phosphorylation site, average normalized abundance values were calculated per iTRAQ channel. Ratios were then obtained for unstimulated and stimulated platelets per protein and subject. Then, the ratio cohorts were compared between subjects.

##### Targeted Protein Quantification by Parallel Reaction Monitoring (PRM)

To validate differential proteins obtained from global quantitative proteomics we used targeted PRM to quantify selected proteins in unstimulated platelets from three healthy controls and the Scott patient. Therefore, we used a U3000 nano-RSLC system online-coupled to an Orbitrap Fusion mass spectrometer, with LC parameters as above. The polysiloxane *m*/*z* 445.1200 was used for lock mass calibration. Target peptides were isolated in the quadrupole with an isolation window of 0.4 *m*/*z* and fragmented using HCD with a collision energy of 30. The AGC target value was set up to 1 × 10^5^ ions, the maximum injection time to 200. MS/MS spectra were acquired in the Orbitrap with a resolution of 30,000. For data analysis we used MS/MS spectra from the label-free quantification data (see above) as well as in-house spectral libraries and Skyline (33).

##### Charge-based Fractional Diagonal Chromatography (ChaFRADIC)for Determination of N-terminal Cleavage Sites

Scott and healthy platelet lysates were diluted 10-fold with ice cold ethanol and stored at −40 °C for 1 h. Afterward, samples were centrifuged at 18,000 × *g* for 30 min at 4 °C. Protein pellets were resuspended into 10 μl 10% SDS under ultrasonication, and diluted to 1% SDS with 0.5 m TEAB containing 20% (v/v) isopropanol. After addition of iTRAQ 8-plex reagents in isopropanol (40 μl), labeling was performed on the protein level for 2 h at 25 °C, in order to block free protein N termini and Lys residues. The eight samples were pooled, and reactions were quenched by addition of 60 mm glycine (10 min), followed by 130 mm hydroxylamine to reverse side reactions of hydroxyl groups (10 min).

For proteolytic digestion, the multiplexed sample was divided into three aliquots of ∼160 μg protein, which were individually subjected to ethanol precipitation as described above. Proteolytic digestion was performed and controlled, as described above. Digestion was stopped with 1% TFA. The peptide mixtures were desalted using C_18_ solid phase tips (SPEC C_18_ AR, 4 mg bed, Agilent Technologies). ChaFRADIC was conducted as previously described (17). Dried pellets were resuspended in 52 μl of SCX solvent A (10 mm KH_2_PO_4_ in 20% acetonitrile, pH 2.7). Peptide samples were separated at a flow rate of 80 μl/min using a U3000 HPLC system with a polysulfoethyl A column (150 × 1 mm; 5 μm particle size; 200 Å pore size; PolyLC), and a tertiary buffer system, *i.e.* solvent A (see above), solvent B (10 mm KH_2_PO_4_, 250 mm KCl in 20% acetonitrile, pH 2.7), and solvent C (10 mm KH_2_PO_4_, 600 mm NaCl in 20% acetonitrile, pH 2.7). The following gradient steps were performed: 100% solvent A for 10 min; followed by a linear ramp from 0–15% solvent B in 9 min; 9 min of 15% B; increase to 30% B over 8 min; 11 min at 30% B; increase to 100% B in 5 min. Five min later, solvent C was increased to 100% in 1 min; 5 min later, solvent A was increased to 100% in 1 min; and the column was equilibrated for 20 min. Five fractions were collected, corresponding to charge states +1, +2, +3, +4 and >+4. These were dried under vacuum and resuspended into 50 mm Na_2_HPO_4_ (pH 7.8). Free N termini of internal peptides, derived from trypsin digestion, were blocked by trideutero (d3)-acetylation to reduce their charge states at pH 2.7. Therefore, trideutero N-hydroxysuccinimide acetate (d3-NHS acetate) was added to a final concentration of 20 mm and, after 1 h at 37 °C, to 30 mm. After 2 h, the reaction was quenched by treatment with 60 mm glycine (10 min), followed by 130 mm hydroxylamine (10 min). Fractions were dried under vacuum and desalted using Poros Oligo R3 reversed-phase material. Desalted fractions were solubilized in 52 μl buffer A, and were individually subjected to rechromatography using the same conditions as above. Because of the acetylation between the first and second dimension SCX fractionations, internal peptides shifted to earlier charge state windows, whereas iTRAQ-labeled and endogenously acetylated N-terminal peptides retained their charge state window and could be enriched.

Fractions were desalted, resuspended in 15 μl of 0.1% (v/v) trifluoroacetic acid, and analyzed by nano-LC-MS/MS using a Q-Exactive Plus online-coupled to a U3000 nano-RSLC system in DDA mode. Survey scans were acquired at a resolution of 70,000, and the top 15 ions were selected for MS/MS at a resolution of 17,500. AGC values were set to 1 × 10^6^ for MS and 2 × 10^5^ for MS/MS scans. A normalized collision energy of 30 was used, and the first fixed mass was set to 100 *m*/*z*. For the +3, +4, and +5 fractions, 10% ammonia solution was placed in front of the ion source. For analysis of the +1 fraction, fragmentation of singly charged peptides was allowed, excluding *m*/*z* values of singly charged ions occurring in previous blank runs. Generated raw data were searched against the human Uniprot database (as above), using Proteome Discoverer version 1.4 and Mascot as search engine, reporter ion quantifier and percolator nodes. Enzyme specificities were set to semi-ArgC, as lysine residues were blocked by iTRAQ labeling on the protein level.

To separate peptides with N-terminal iTRAQ label from those with endogenous N-acetylation, a two-step search strategy was performed. First, data were searched with iTRAQ-8plex (+304.2053 Da) as fixed modification at lysine and peptide N terminus. Second, iTRAQ-8plex of lysine and acetylation of N termini (+42.0105 Da) were set as fixed modifications. In both cases, carbamidomethylation of cysteine (+57.0214 Da) was selected as fixed, whereas oxidation of methionine (+15.9949 Da) was used as variable modification. Mass tolerances were set to 10 ppm for MS and to 0.02 Da for MS/MS. Identified peptides were filtered for high confidence corresponding to an FDR <1% at the PSM level, and a search engine rank of 1. The reporter ion quantifier node was adjusted according to the search settings. Quantification of N-terminal peptides across samples and conditions was performed as described for the phosphoproteome analysis, including grouping, normalization, and rationing.

##### Identification of Calpain Substrates and Cleavage Sites

Hypothesizing that calpain-induced cleavage in activated Scott platelets is altered, we aimed to identify potential calpain substrates. Thus, for the identification of calpain cleavage sites and substrates in control and Scott platelets we pursued a two-step strategy.

First, to define a putative calpain consensus motif but not identify substrates, we digested platelet lysates *in vitro* with calpain, at a protein/enzyme ratio of 1:10 (w/w) using FASP to remove undigested protein. Calpain treatment was performed in 50 mm Tris-HCl, 100 mm NaCl, and 3 mm CaCl_2_ pH 7.45 (30 min at 30 °C). Vehicle controls were run in the absence of calpain. Generation of proteolytic peptides was confirmed by monolithic HPLC (23), showing an extensive peptide pattern for the calpain-treated platelets, whereas the control showed no peptide signals. Thus, the calpain samples were analyzed by nano-LC-MS/MS, and data were processed as above, but with enzyme specificity set to “none” to identify peptide sequences that were generated upon calpain cleavage of denatured proteins. Icelogo (http://iomics.ugent.be/icelogoserver/main.html) was used to visualize potential consensus cleavage patterns considering four amino acids N-terminal and C-terminal of cleavage sites, *i.e.* positions P4-P4′ according to Schechter and Berger (34).

Second, control platelets from healthy donor (1.5 × 10^8^/ml) were activated with ionomycin (10 μm) in the presence or absence of calpain inhibitor, calpeptin (200 μm) for 30 min, or with the proapoptotic agent, ABT-737 (10 μm) in the presence or absence of caspase inhibitor QVD-OPh (20 μm) 60 min at 37 °C, and lysed as above. These samples were used for N-terminal quantitative ChaFRADIC as described above, in duplicate.

##### Deposition of Raw Data

Raw data and Proteome Discoverer search results are deposited in the ProteomeXchange repository (34) dataset identifiers PXD002883 and 10.6019/PXD002883.

##### Experimental Design and Statistical Rationale

##### Sample Sets

Purified washed platelets from a healthy control donor (5 × 10^8^/ml) and Scott patient (5 × 10^8^/ml), left untreated or activated with thrombin, thrombin/convulxin or ionomycin were used for (1) global label free quantification (only untreated conditions), (2) combined iTRAQ global proteome and phosphoproteome quantification (all samples), and (3) iTRAQ-based quantification of N-terminal peptides. Owing to the limited amount of available sample, (1) and (2) were performed using one technical replicate, each. Notably, the four different conditions in the global iTRAQ proteome data plus the label free analysis can be considered as five technical replicates. For (3) we conducted three technical replicates of the entire workflow following protein labeling and precipitation as we expected higher variations of the complex ChaFRADIC procedure for which we only required 20 μg of protein per replicate and condition (*i.e.* iTRAQ channel).

Furthermore, the same samples were used for Western blot and flow cytometry (1.5 × 10^8^/ml). Additionally, for flow cytometry experiments platelets from two independent healthy control donors were used. For whole blood flow experiments, blood from the only Scott patient and from four healthy control donors was used.

For the identification of potential calpain substrates, platelets from a healthy control donor (1.5 × 10^8^/ml) were treated with ionomycin in the presence or absence of calpain inhibitor, calpeptin for 30 min or for 1 h with ABT-737, as well in presence or absence of calpain inhibitor, calpeptin. These samples were used for quantitative N-terminal peptide analysis with ChaFRADIC, using 2 technical replicates as above.

For validation of differential proteins using PRM, platelets from three healthy control donors (two from a completely different set of samples) and platelets from the only Scott patient were used.

##### Analysis and Statistics

For the global proteome, interindividual variation over all proteins was estimated by separate analysis of samples (unstimulated platelets) from pairs of healthy subjects; after log2 transformation this gave a mean S.D. of 0.158 (range 0.107–0.200, *n* = 4). Boundaries for relevant changes in patient samples were set at 2 × S.D. (2 × 0.158) in comparison to median abundance ratios, to determine up- or downregulation per protein. For interpretation of iTRAQ phosphopeptide ratios, interindividual variation over all phosphopeptides was estimated from separate analysis of resting platelets from pairs of subjects; after log2 transformation, this gave a mean S.D. of 0.385 (range 0.337–0.420, *n* = 4). Boundaries for relevant changes were again set at 2 × S.D. (2 × 0.385) in comparison to median abundance ratios, for up- or downregulation per phosphopeptide. For comparison of iTRAQ ratios of N-terminal peptides, mean S.D. (log2 transformed) of four sample sets was 0.515; again cut-off values for up- and downregulation were set at 2 × S.D. (2 × 0.515) in comparison to median abundance ratios.

Data from functional tests were expressed as means ± S.E. Statistical significance of differences between groups was determined using a 2-way ANOVA. *p* values <0.05 considered significant. Lists of ratios were compared by the Dunnett's test for multiple groups.

## RESULTS

### 

#### 

##### Phenotypic Analysis of Scott Syndrome Platelets

**Fig. 2.**
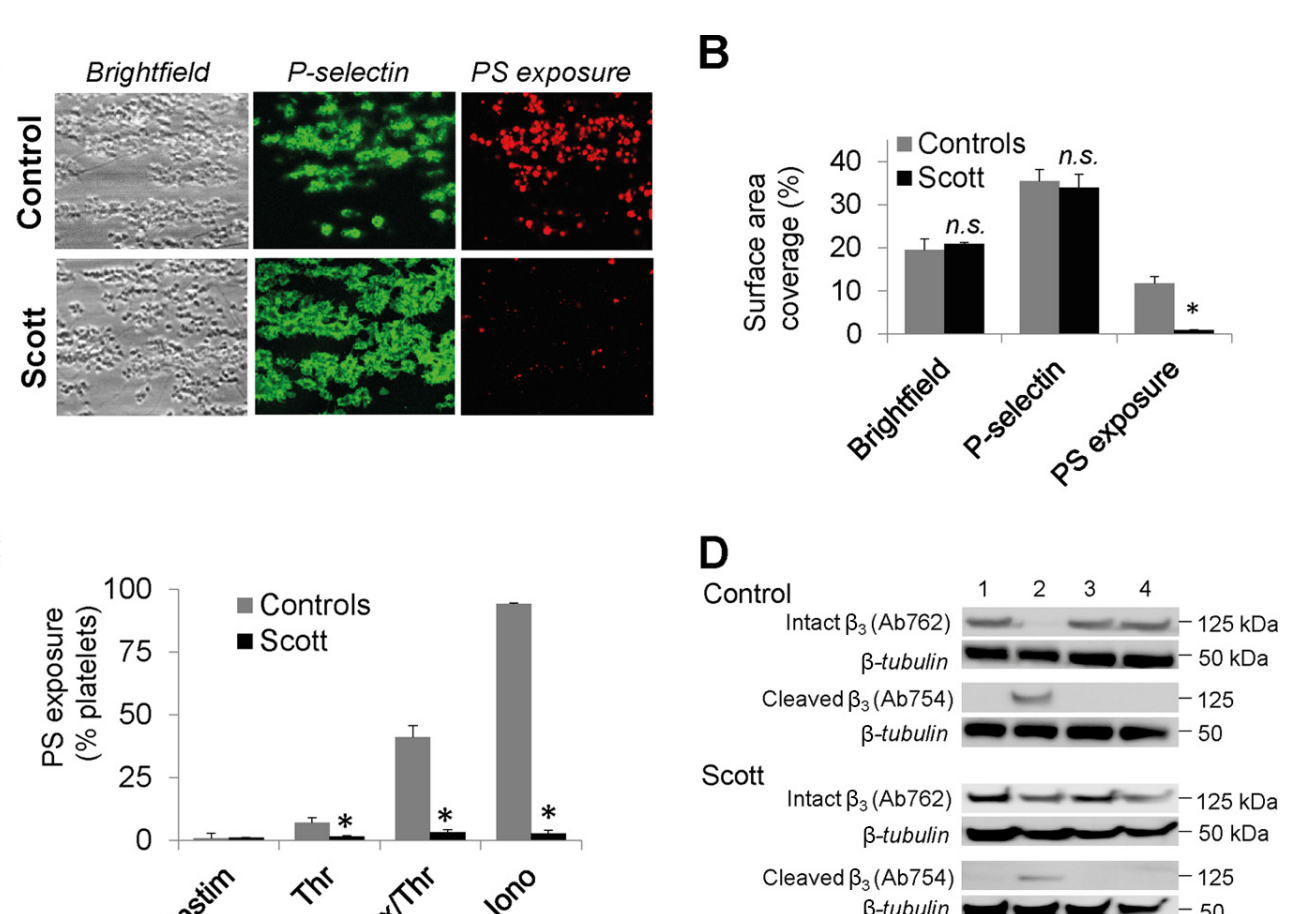
**Phenotypic analysis of activated Scott platelets.** *A–B*, Unchanged platelet deposition and P-selectin expression, but impaired PS exposure in collagen-dependent thrombus formation. *A*, Thrombi formed by whole-blood perfusion from a control subject or Scott patient were stained with FITC-anti-P-selectin mAb and AF647-annexin A5. Representative brightfield and fluorescence images (200 × 165 μm). *B*, Surface-area-coverage of platelet deposition, P-selectin expression and PS exposure. *C*, Impaired PS exposure of Scott platelets stimulated with Ca^2+^-elevating agonists. Washed platelets (1.5 × 10^8^/ml) were activated with thrombin (4 nm), thrombin/convulxin (100 ng/ml, 4 nm), or ionomycin (10 μm). Exposure of PS was determined after 30 min of activation. *D*, Reduced calpain-dependent integrin β_3_ cleavage of Scott platelets. Washed platelets (5 × 10^8^/ml) were preincubated with vehicle or one of the calpain inhibitors, calpeptin (200 μm), or MDL-28170 (200 μm), and then stimulated with thrombin/convulxin (100 ng/ml, 4 nm) for 30 min. Western blots were stained for intact integrin β_3_ with Ab762 or for cleaved integrin β_3_ with Ab754. Blots were reprobed with anti-α-tubulin Ab as protein loading control. Lane 1, resting platelets; lanes 2–4, thrombin/convulxin stimulated platelets: lane 2, vehicle treatment; lane 3 calpeptin treatment; lane 4, MDL-28170 treatment. Representative for 3 experiments. Means ± S.E. (*n* = 3–4), *p < 0.05 compared with controls (2-way ANOVA).

To confirm the complex altered phenotype of Scott platelets, we analyzed whole blood and isolated platelets from a Scott syndrome patient with two likely disruptive mutations in the anoctamin-6 alleles. In comparison to blood from healthy control subjects, perfusion of the patient's blood over collagen resulted in normal formation of thrombi and normal P-selectin expression (Fig. 2*A*, 2*B*), indicating that platelet integrin activation and α-granule secretion were unchanged. However, with the patient's blood PS exposure was greatly reduced, leaving only small patches of exposed PS on some platelets. Earlier, we have established that this near complete failure of PS exposure is accompanied by an inability of the platelets to swell and form a balloon-like morphology (35). Similarly, isolated platelets from the Scott patient were greatly impaired in PS exposure in response to strong Ca^2+^-mobilizing agonists, such as thrombin/convulxin (stimulating thrombin and collagen receptors) and to the Ca^2+^-ionophore ionomycin (Fig. 2*C*). With platelets from control subjects, PS exposure with these agonists was high, *i.e.* in 41 ± 5% and 94 ± 1% of the platelets, respectively (means ± S.E., *n* = 3). As reported before (6), thrombin alone induced only 7 ± 2% PS exposure in control platelets, whereas this fraction was reduced to 2 ± 1% in Scott platelets. Additional evidence for decreased calpain activity in thrombin/convulxin-stimulated Scott platelets came from Western blotting of platelet samples. Using an antibody recognizing the intact cytosolic N-terminal site of the integrin β_3_-chain (Ab762) and an antibody recognizing a calpain-cleaved N-terminal site in the β_3_-chain (Ab754) (11), it appeared that Scott platelets showed a relatively high staining with Ab762 and a weaker staining with Ab754 (Fig. 2*D*). The Ab754 staining was completely antagonized with two calpain antagonists, calpeptin and MDL-28170, thus demonstrating calpain-mediated protein cleavage of the integrin.

##### Quantitative Assessment of the Global Proteome of Scott Platelets

To assess the platelet protein composition and phosphorylation state, we compared well-purified washed Scott and control platelets (contamination with erythrocytes <1:15,000, with leukocytes <1:20,000). Freshly isolated platelets stayed unstimulated or were stimulated with high concentrations of thrombin, thrombin/convulxin, or ionomycin for a prolonged time of 30 min, *i.e.* at conditions comparable to those of the measurements of PS exposure. Samples were lysed, digested with trypsin, and labeled with 8-plex iTRAQ reagents for global and phospho-proteome analysis (Fig. 1), whereas for N-terminal ChaFRADIC samples were iTRAQ labeled on the protein level, multiplexed and digested.

**Fig. 3.**
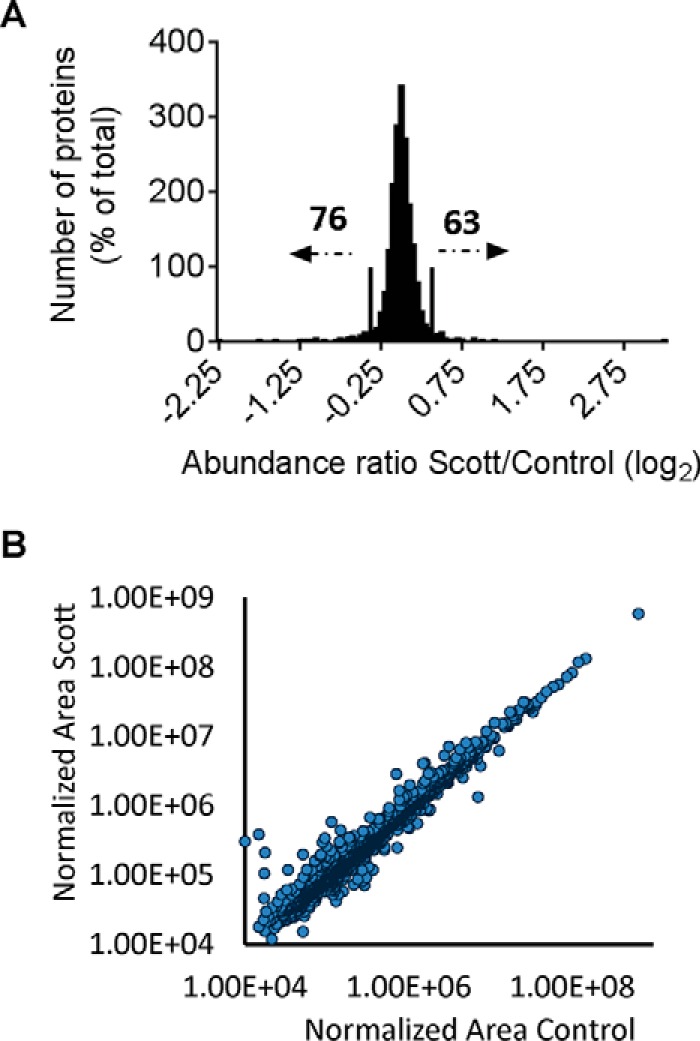
**Moderate changes in quantitative proteome of Scott platelets.** *A*, Ratios of iTRAQ-labeled fragments of 1961 unique proteins were quantified in highly purified Scott and control platelets (no stimulation). Ratios of Scott/control platelets were log2 transformed and plotted as a function of the number of proteins. Ratios were considered to be relevant if > or < 2 × 0.158 from the median (log2 transformed). Of the 1961 proteins, 70 were assigned as downregulated and 64 as up-regulated in Scott platelets. For pairs of controls subjects (*n* = 4), this yielded by default ∼45 proteins in either category. *B*, Correlation of normalized area values of 1,435 unique proteins from label-free global proteome analysis of Scott and control platelets.

Using iTRAQ we quantified 2278 unique proteins with at least two unique peptides (supplemental Data S1). This corresponded to ∼50% of the estimated platelet proteome (13). Considering a minimum deviation of 2 × S.D. (log2 transformed) for relevant changes 58 (2.5%) and 46 (2.0%) of proteins were potentially down- or up-regulated in Scott platelets, respectively (Fig. 3*A*). Owing to potential issue of ratio compression in iTRAQ, we also performed label-free proteome analysis (28, 29) of the unstimulated Scott and control platelets. This label-free analysis resulted in the quantification of 1435 unique proteins with at least two unique peptides (supplemental Data S1). This data also pointed to a high similarity of the protein distribution pattern in the Scott and control platelets (Fig. 3*B*). Overall, the relative abundance of individual proteins was comparable to earlier findings (13). Importantly, the label-free analysis confirmed most changes in abundance of the patient platelets obtained from iTRAQ ratios (supplemental Table S1).

The protein with the strongest downregulation in Scott platelets was anoctamin 6 (gene *ANO6*) (supplemental Table S1). The proteins with the strongest decrease in Scott platelets further include signaling and adapter proteins (genes *S100A8, S100A9*) implicated in Ca^2+^ regulation, and several uncommon secretory proteins (*genes PZP, MPO, AGT, PON1*). On the other hand, the most increased proteins in Scott platelets (supplemental Table S1) surprisingly comprised the membrane channel protein aquaporin-1 (gene *AQP1*). Most likely because of its apparent absence in control platelets, aquaporin-1 could only be quantified in the iTRAQ proteome with one regulated peptide, but it was highly up-regulated in the label-free proteome with two peptides. The list of up-regulated proteins also comprises proteins involved in transcription and translation (*RPLP2, RPLP0*); and proteins implicated in platelet activation (*CD36, PRKAR2B*) and PS binding (*ANXA5*).

**Fig. 4.**
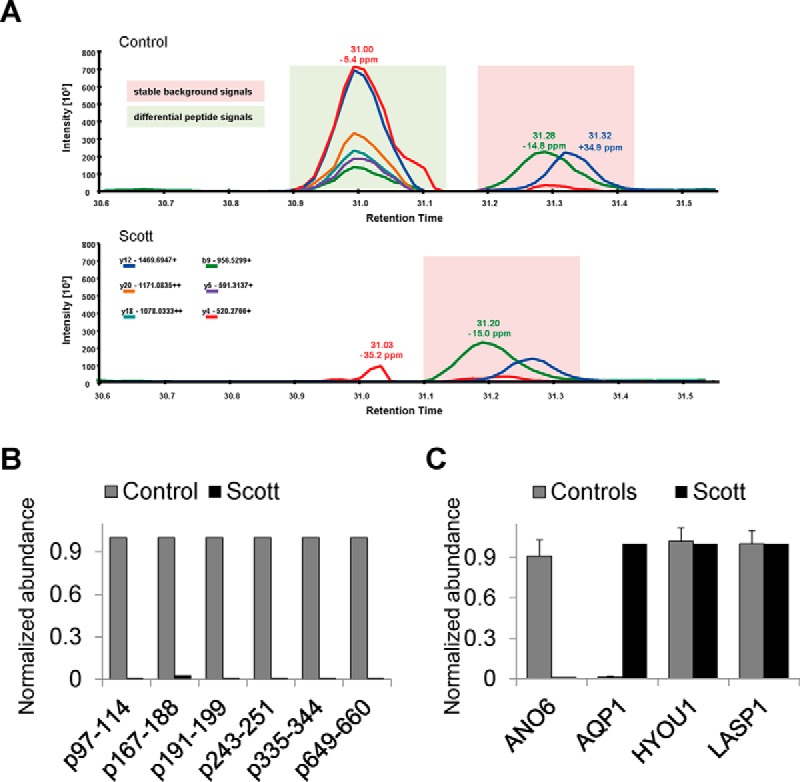
**High-resolution parallel reaction monitoring (PRM) for validation.** *A*, Representative PRM analysis of the anoctamin-6 peptide ^167^VLSVDESIIKPEQEFFTAPFEK^188^ in control and Scot platelets. Note that the correct peptide at retention time of 31.0 min was present in the control but not patient sample, whereas the background peaks at 31.2 min show similar patterns and intensities. Because of the mass deviation of −35.2 ppm and the incomplete peak pattern, the residual signal at 31.03 min in the Scott sample can be assigned to noise. *B*, Normalized abundance of 6 peptides from anoctamin-6, representing different parts of the protein, in Scott platelets and platelets from three controls. Peptides analyzed were ^97^QAYESNLICHGLQLEATR^114^, ^167^VLSVDESIIKPEQEFFTAPFEK^188^ (residual Scott signal can be attributed to noise, see above), ^191^MNDFYIVDR^199^, ^243^AAFPLHDCK^251^, ^335^EVCHPDIGGK^344^, and ^649^WEQDYHLQPMGK^660^. *C*, Normalized abundance of peptides from anoctamin-6 (*ANO6*, 6 peptides), aquaporin 1 (*AQP1,* 2 peptides), hypoxia up-regulated protein 1 (*HYOU1*, 4 peptides), and LIM and SH3 domain protein 1 (*LASP1*, 5 peptides). Intensities for the patient sample were set to 1.0 and compared with 3 control samples (except for *ANO6*).

As independent validation of the *ANO6* and *AQP1* quantification, we also followed a more sensitive targeted approach. Using parallel reaction monitoring (PRM) as a high resolution MS/MS technique, we quantified peptides/proteins of interest directly in whole platelet digests. Thus, in platelets from three healthy controls and the Scott patient, we quantified six peptides for *ANO6*, two peptides from *AQP1* and, as indifferent control proteins, ≥two peptides from hypoxia up-regulated protein 1 (*HYOU1*) and LIM and SH3 domain protein 1 (*LASP1*). We selected aquaporin-1 as its up-regulation might be compensatory for the anoctamin-6 ion channel deficiency, which would be in agreement with the recently established role of aquaporin water channels in platelet morphological changes such as ballooning (35). For anoctamin-6, PRM indicated that the residual signal in the Scott samples could be attributed to noise, because of clear deviations in mass and peak pattern (Fig. 4*A*). Hence, the abundance level of anoctamin-6 in Scott platelets was below the detection limit, *i.e.* at least 50–100 times lower than in control platelets (Fig. 4*B*). Aquaporin-1 could not be detected in any of the three control samples, whereas it was clearly present in the Scott patient platelets (Fig. 4*C*). As expected, abundance levels of the *HYOU1* and *LASP1* proteins were highly similar in all platelet preparations. To confirm, that the increased aquaporin-1 levels in Scott platelets do not derive from potential erythrocyte contaminations, we compared our proteomic data with the erythrocyte proteome from Wiśniewski *et al.* (36) and thus can exclude that the presence of aquaporin-1 in Scott platelets may be explained by erythrocyte contamination. Altogether, these data pointed to mostly moderate changes in the global proteome of Scott platelets with exception of *ANO6* and *AQP1* proteins.

##### Changes in Phosphorylation Profile of Activated Scott Platelets

**Fig. 5.**
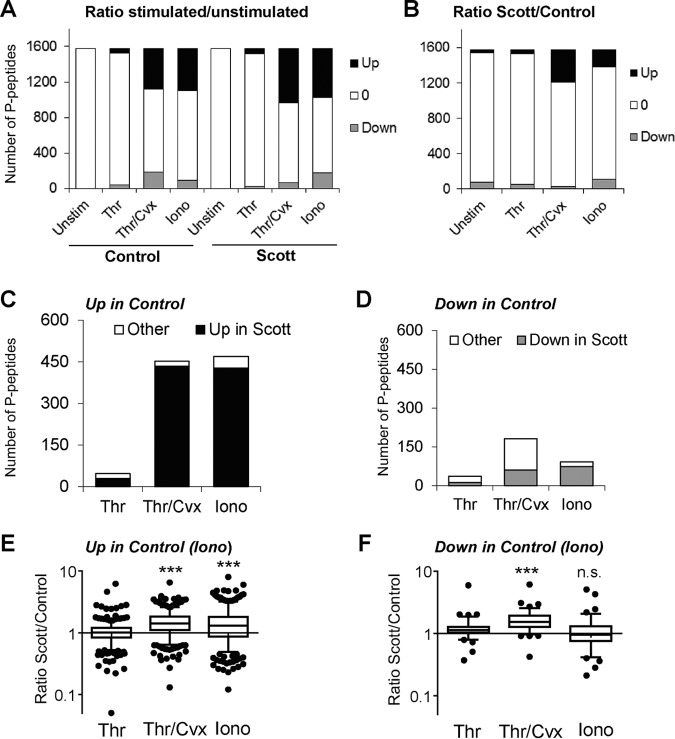
**Phosphoproteomic changes in Scott platelets subjected to strong activation.** iTRAQ-based quantification of (un)stimulated control and Scott platelets. Stimulation for 30 min was with thrombin (Thr), thrombin/convulxin (Thr/Cvx), or ionomycin (Iono). Abundance ratios per identified phosphopeptide were classified as changed when outside the range (log2 transformed) > or < 2 × 0.385 compared with the median of unstimulated cells (see Methods). 0, stable. *A*, Numbers of up- and downregulated phosphopeptides in response to agonists. *B*, Numbers of up- and downregulated phosphopeptides in (stimulated) Scott platelets *versus* control platelets. *C*, *D*, Numbers of up- and downregulated phosphoproteins in control platelets with similar change in Scott platelets. *E*, *F*, Box plots of Scott/control ratios of all phosphopeptides, assessed as up-regulated and downregulated in ionomycin-stimulated control platelets, per agonist condition. ***, Significant with Dunnett's test for multiple groups.

After iTRAQ labeling and TiO_2_ enrichment of the pooled Scott and control platelets, we quantified 1566 phosphopeptides, corresponding to 709 unique proteins (supplemental Data S2). Per identified phosphopeptide, again we considered iTRAQ ratios as relevant changes, when deviant outside the range of 2 × S.D. (log2 transformed) in distribution curves from control platelets. Long-term (30 min) stimulation of control or patient platelets with thrombin resulted in no more than small changes in phosphorylation pattern (Fig. 5*A*). This is in agreement with earlier work showing that protein (tyrosine) phosphorylation in thrombin-stimulated platelets peaks within 1 min (37), and is mostly returned to resting levels after 10 min (38).

In contrast, platelet stimulation with thrombin/convulxin or ionomycin dramatically increased the overall phosphorylation state. Comparing ratio values of Scott to control platelets, these agonists evoked an increased phosphorylation in 404 and 206 peptides, respectively (Fig. 5*B*). Conversely, the same agonists evoked a decreased phosphorylation in 25 and 105 peptides, respectively. On the other hand, we assessed that 90% of the phosphopeptides that were up-regulated in activated control platelets were also increased in the patient's platelets (Fig. 5*C*). For the downregulated phosphopeptides, lower percentages were obtained (Fig. 5*D*). Comparative analysis for the up-regulated phosphopeptides indicated a significant overall increase in the patient's platelets after stimulation with thrombin/convulxin or ionomycin (Fig. 5*E*) as compared with the control. Also for the downregulated phosphopeptides, relative abundance levels in the Scott platelets were significantly increased after thrombin/convulxin (Fig. 5*F*). Jointly, these data pointed to an overall increased phosphorylation state of the activated Scott platelets, especially after stimulation with thrombin/convulxin.

Assignment tabling indicated that mostly Scott proteins of the following functional classes were increased in phosphorylation: cytoskeleton-linked; signaling and adapter proteins; small GTPases and regulators; protein kinases and phosphatases; and membrane receptors and channels (Table I). Zooming in to proteins with the strongest changes (supplemental Table S2) revealed decreased phosphorylation in Scott platelets of several signaling and adaptor proteins (genes *PDE3A, ENSA, NCK2, STIM1*), proteins implicated in PS exposure (3, 39), *i.e.* BLC2 (*BNIP2*). The highest increases in phosphorylation of Scott platelets were seen for several cytoskeletal-linked proteins (*SEP6, DBNL, PLEK, TNS1, PDLIM7, MYH9, TNL1*), and the small GTPase activator ARHGAP6. These lists contain considerable overlap with the phosphoproteome changes reported for human platelets activated with thrombin or oxidized phospholipids (16). Together, these data suggested that the altered phosphorylation pattern of Scott platelets upon strong stimulation involves multiple cytoskeletal-linked proteins and modulators of PS exposure.

**Table I:** Overview of differences in phosphoproteome of Scott platelets compared to healthy control platelets. 1566 quantified phosphosites corresponding to 709 proteins were assigned to 21 platelet function classes. Numbers or percentages are shown per function class of phosphopeptides with decreased or increased phosphorylation in activated Scott platelets in comparison to control platelets. Changes in abundance were considered to be relevant for phosphopeptide ratios <0.590 or >1.748

Functional class	Thr		Thr/Cvx		Iono		total p-peptides
	Down	Up	Down	Up	Down	Up	
Cytoskeleton actin-myosin	2%	3%		35%	7%	20%	146
Cytoskeleton intermediate					33%		6
Cytoskeleton microtubule	1%	4%	3%	36%	7%	7%	73
Cytoskeleton receptor-linked	1%	5%	1%	26%	10%	13%	111
Endosomal proteins				29%			7
ER & Golgi proteins			8%	17%	8%	13%	24
Glucose metabolism			13%	13%	6%	6%	16
Lysosome & peroxisome proteins				67%		67%	3
Membrane & protein trafficking	3%	4%	2%	25%	5%	7%	102
Membrane receptors & channels	3%	3%	1%	22%	9%	13%	184
Mitochondrial proteins				38%	13%	13%	8
Other metabolism	4%	9%		11%	9%	15%	46
Other nuclear proteins		2%		26%	2%	7%	43
Phospholipid regulation				100%		100%	1
Proteasome	3%	5%	2%	14%	2%	9%	58
Protein kinases & phosphatases	1%	3%	3%	24%	6%	15%	151
Secretory proteins	5%		5%	11%		11%	19
Signalling & adapter proteins	5%	3%	2%	28%	6%	17%	259
Small GTPases & regulators	3%	4%	1%	29%	11%	12%	166
Transcription & translation	2%	5%		26%		14%	58
Unknown	3%	4%	1%	26%	5%	13%	80
Total	42	54	25	403	105	206	1564

##### Identification of Platelet Proteins Cleaved by Calpains or Caspases

Platelet PS exposure is accompanied by activation of intracellular proteases, such as calpain isoforms in platelets that are stimulated with strong Ca^2+^-elevating agents (11) or with Ca^2+^-independent caspases in apoptotic platelets (39). As Western blots indicated that calpain-mediated integrin cleavage is impaired in Scott platelets, we set to determine the substrates of this protease in platelets.

**Fig. 6.**
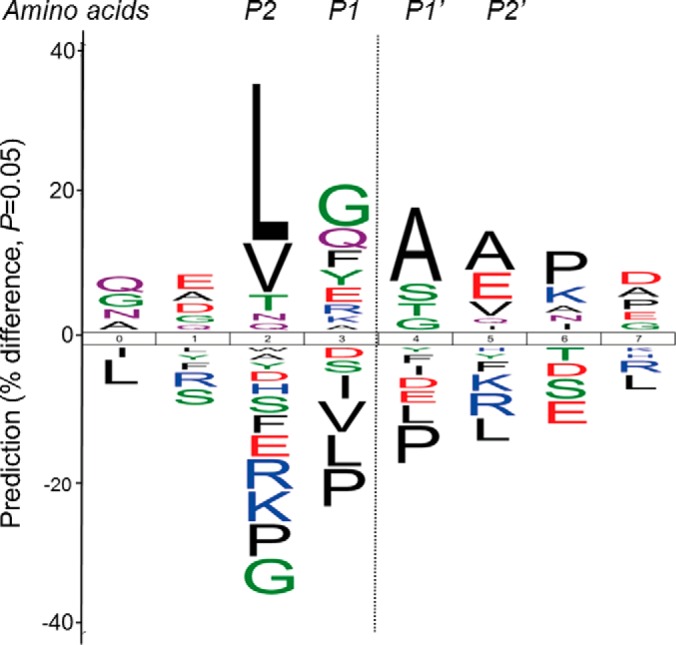
**Experimentally predicted calpain cleavage motif.** LC-MS analysis of an *in vitro* platelet digest identified 2224 high confident calpain-generated peptides, corresponding to 375 unique proteins. Icelogo representation of P4-P4′ positions (4 amino acids upstream to 4 amino acids downstream of the cleavage site) of the obtained peptide data to visualize the experimentally obtained calpain cleavage consensus motif. Large size characters indicate amino acids with a large positive or negative predictive contribution to calpain cleavage.

To identify calpain substrates in platelets we combined two different approaches. First, as reports on calpain cleavage specificity were controversial, we aimed at defining a consensus motif. Therefore, we digested lysates from control platelets *in vitro* with purified calpain to first obtain insights into the general cleavage pattern, rather than to identify *in vivo* substrates. The digested sample was analyzed by LC-MS, followed by database searches without enzyme specificity. This resulted in a list of 2224 unique peptides at 1% FDR corresponding to 375 proteins. These peptides were used to determine a cleavage pattern with IceLogo, identifying amino acids overrepresented in the proximity of the cleavage site (Fig. 6).

**Fig. 7.**
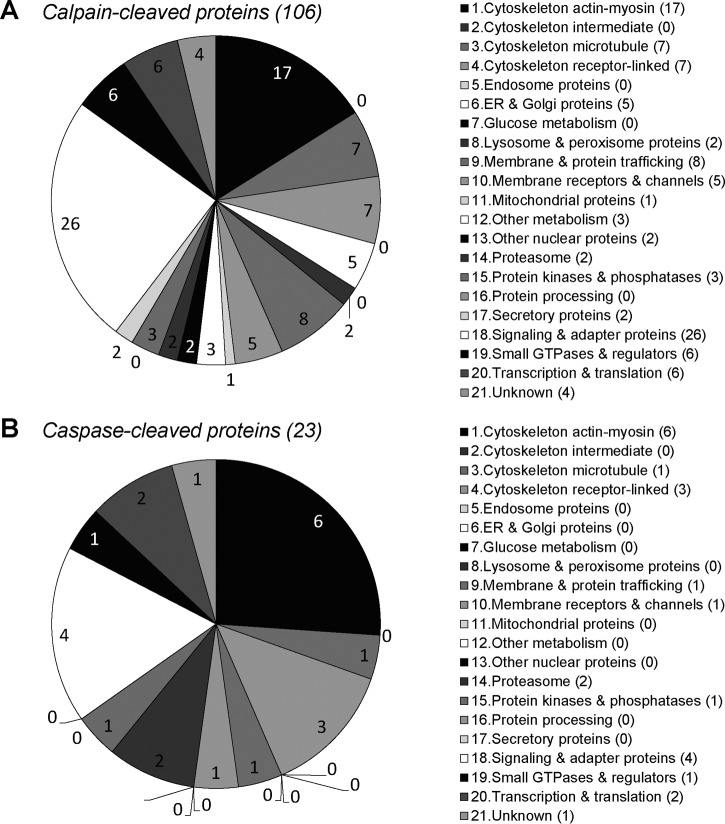
**Class distribution of calpain- and caspase-cleaved proteins in platelets.** Control platelets were stimulated with ionomycin (30 min) or ABT-737 (60 min) in the presence or absence of calpain inhibitor calpeptin or caspase inhibitor QVD-OPh (see supplementary Table S1). *A*, Distribution of 106 identified calpain-cleaved proteins over platelet function classes (>3-fold increased cleavage with ionomycin and >50% reduction with calpeptin). *B*, Distribution of 23 identified caspase-cleaved proteins over platelet function classes (>3-fold increased cleavage with ABT-737 and >50% reduction QVD-OPh).

Second, we used our recently developed ChaFRADIC workflow for determination of neo-N-terminal peptides formed *in vivo* upon proteolytic cleavage (17, 18). Thus, intact control platelets were stimulated with Ca^2+^-mobilizing ionomycin or the proapoptotic agent ABT-737 in the presence or absence of calpain inhibitor calpeptin or caspase inhibitor QVD-Oph (11). Quantitative analysis of the platelet lysates by ChaFRADIC revealed 227 neo-N-terminal peptides with a >3-fold increased levels in ionomycin-stimulated platelets, 180 of which were inhibited by calpeptin, but not by QVD-Oph (supplemental Table. S3*A*). These 180 calpeptin-sensitive neo-N-terminal peptides corresponded to 106 proteins. The *in vivo* list includes proteins previously found to be cleaved in PS-exposing platelets, namely Src kinase (*SRC*) and talin-1 (*TLN1*) (11). Interestingly, many of the proteins seemed to be cleaved by calpain on multiple sites, *e.g.* caldesmon (*CALD1*), a calpain-1 subunit (*CAPNS1*), kindlin-3 (*FERMT3*), filamin-A (*FLNA*), myosin-9 (*MYH9*), talin-1 (*TLN*), vasodilator-stimulated phosphoprotein (*VASP*), and zyxin (*ZYX*) that are cleaved at four or more positions. The majority of the 106 proteins could be assigned to the following functional classes: cytoskeleton-linked, membrane and protein trafficking, or signaling and adapter proteins (Fig. 7*A*).

In the apoptotic ABT-737-treated platelets, we identified 45 neo-N-terminal peptides with a >3-fold increase, 23 of which were inhibited by QVD-Oph, but not calpeptin (supplemental Table S3*B*). The corresponding 23 proteins, in part overlapped with the calpain-cleaved proteins—*e.g.* α-actinin-1 (*ACTN1*), *FERMT3*, glycoprotein Ibα (*GPIBA*), *FLNA*, leucine-rich repeat flightless-interacting protein 1 (*LRRFIP1*), *MYH9*, nexilin (*NEXN*), and Pyk2 (*SKAP2*)—but were caspase-cleaved at a different position. The 23 identified caspase substrates mostly categorized as cytoskeletal-linked or signaling and adaptor proteins (Fig. 7*B*). Together, these results revealed a pattern of distinct cleavage sites by calpains and caspases in platelets, in particular of cytoskeletal-associated and signaling proteins.

##### Changes in N-terminal Proteome of Activated Scott Platelets

**Fig. 8.**
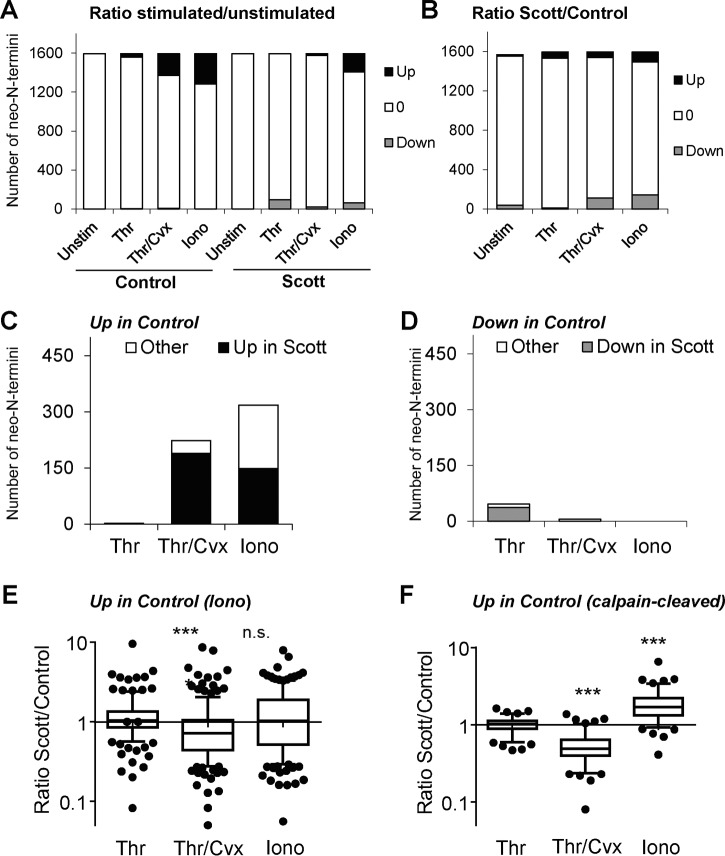
**Changes in N-terminal peptides in Scott platelets subjected to strong activation.** iTRAQ-based quantitative N-terminal ChaFRADIC was used to quantify changes in N-terminal peptide abundance between (un)stimulated control and Scott platelets, in order to identify alterations in proteolytic activity. Stimulation for 30 min was with thrombin (Thr), thrombin/convulxin (Thr/Cvx), or ionomycin (Iono). Abundance ratios per identified peptide were classified as changed when outside the range (log2 transformed) > or < 2 × 0.504 compared with the median of unstimulated cells. *A*, Numbers of up- and downregulated N-terminal peptides in response to agonists. *B*, Numbers of up- and downregulated peptides in (stimulated) Scott platelets *versus* control platelets. *C*, *D*, Numbers of up- and downregulated N-terminal peptides in control platelets with similar change in Scott platelets. *E*, Box plot of Scott/control ratios of all 318 N-terminal peptides assessed as up-regulated in ionomycin-stimulated control platelets, per agonist condition. *F*, Similar box plot, but for 37 confirmed calpain-cleaved N-terminal peptides per agonist condition. ***, Significant with Dunnett's test for multiple groups.

**Fig. 9.**
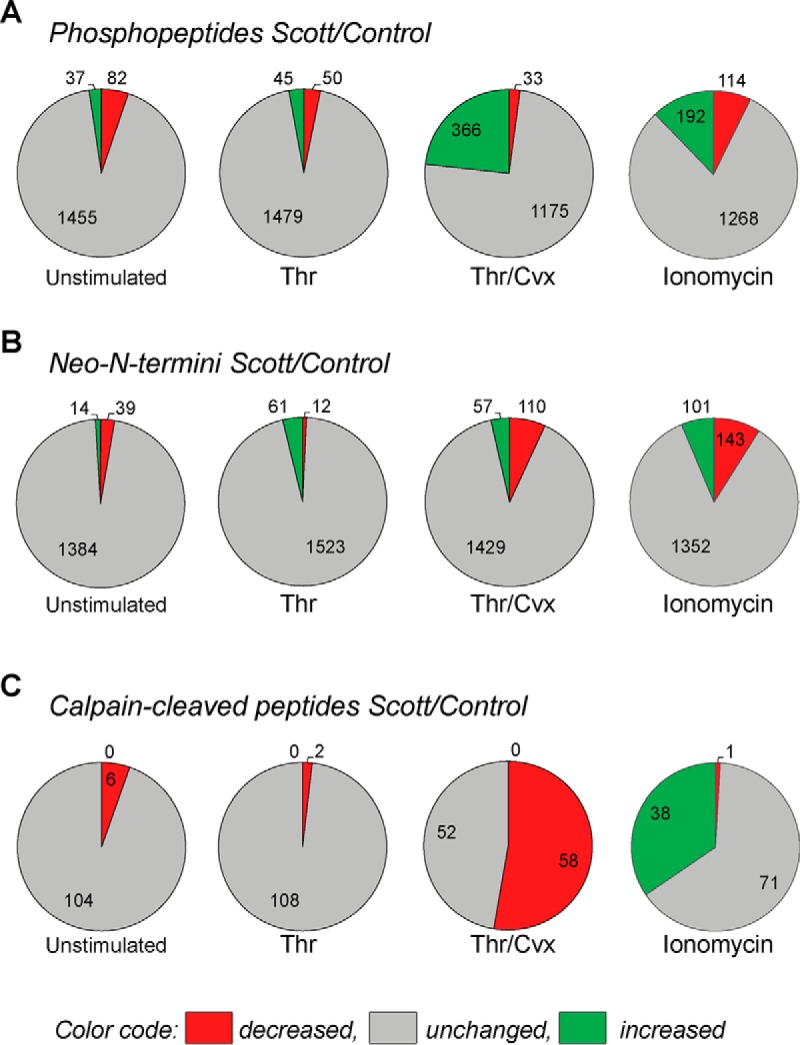
**Altered protein phosphorylation and proteolytic cleavage in activated Scott platelets.** Samples of Scott and control platelets were left unstimulated, or were stimulated for 30 min with thrombin (Thr), thrombin/convulxin (Thr/Cvx) or ionomycin (Iono). Overview per agonist condition: *A*, changes in all 1566 phosphopeptides for (un)stimulated Scott platelets *versus* control platelets; *B*, changes in all 1596 N-terminal peptides for (un)stimulated Scott platelets *versus* control platelets; *C*, changes in 110 N-terminal peptides identified as calpain-cleaved for (un)stimulated Scott platelets.

Next, we conducted quantitative N-terminal ChaFRADICto follow aberrant calpain activity in Scott platelets. Thus, also platelets from the Scott patient and control subject were analyzed by quantitative N-terminal ChaFRADIC. We quantified 1596 N-terminal peptides between Scott and control platelets that were either unstimulated, or stimulated with thrombin, thrombin/convulxin or ionomycin (supplemental Data S3). Considering again a deviation of 2 × S.D. (log2 transformed) in distribution curves from control platelets as relevant changes, we found that only thrombin/convulxin and ionomycin caused major changes in abundance of N-terminal peptides, both in control and Scott platelets (Fig. 8*A*). The same agonists also induced major changes in the ratios of N termini in Scott *versus* control platelets (Fig. 8*B*). In control platelets stimulation induced up-regulation of 2 (thrombin), 223 (thrombin/convulxin), and 318 (ionomycin) N-terminal peptides, respectively, the majority of which was also increased in the Scott platelets (Fig. 8*C*). Markedly, taken together all 318 up-regulated N termini, the extent of up-regulation was significantly decreased in thrombin/convulxin stimulated Scott platelets (Fig. 8*E*). When reducing this list from 318 to 37 up-regulated N-terminal peptides with confirmed cleavage by calpain, as derived from our consensus sequence and inhibitor data, we found that calpain cleavage-mediated up-regulation was significantly reduced in Scott platelets after stimulation with thrombin/convulxin, whereas it was more pronounced after stimulation with ionomycin (Fig. 8*F*). Plotting of the altered N-terminal peptides in Scott *versus* control platelets indicated that more were decreased in the patient, such in contrast to the higher numbers with increased phosphorylation of many phosphopeptides (Fig. 9*A*–9*B*). Concerning the calpain-cleaved N termini, thrombin/convulxin stimulation led to an overall decrease, whereas ionomycin stimulation led to an increase (Fig. 9*C*). Overall, these data pointed to a reduced calpain activity in the Scott platelets when stimulated with thrombin/convulxin, and an increased activity after ionomycin stimulation. The latter difference was surprising, but can be explained by the protection of Scott platelets to membrane dysregulation during swelling and PS exposure at the high intracellular Ca^2+^ concentration reached with ionomycin, which may prolong the calpain activity before the cells die.

Interestingly, the top-15 of decreased N-terminal peptides in the Scott patients (arranged according to ratios obtained with thrombin/convulxin) showed in majority (1) a cleavage pattern with two or more of our consensus amino acids for calpain cleavage and (2) a complete inhibitory effect of calpeptin, as established for control platelets (supplemental Table S4*A*). This confirmed that these N-terminal peptides were generated upon calpain cleavage. The top-15 of decreased neo-N-terminal peptides in the Scott patient included cytoskeleton-linked proteins (genes *DBLN, DNM1L, MAPRE1, NEXN, SMIM1, TLN1,* and *ZYX*) and key regulatory signaling proteins (*DOK3, FYB, GSK3B, PDE3A, VASP*). In contrast, the top-15 of *increased* N termini concerned a variety of proteins with cleavage sites which were not inhibited by calpeptin (supplemental Table S4*B*). Hence, these cleavage sites are unlikely to be calpain-mediated. Collectively, also this analysis showed a decreased calpain-dependent formation of N termini in convulxin/thrombin-stimulated Scott platelets. Interestingly, the majority of the identified calpain cleavage sites differ from those in previous lists of N termini in human platelets (40), likely because these were obtained from unstimulated, stored platelets rather than from activated platelets, as in the present article.

## DISCUSSION

Here we provide a first comprehensive quantitative analysis of the protein composition, protein phosphorylation state and protein cleavage pattern of isolated primary human cells, *i.e.* well-purified blood platelets. By applying these sensitive proteomics techniques to platelets of a patient with the congenital Scott syndrome with mutations in the *ANO6* gene, and a complex altered phenotype (deficiency in Ca^2+^-dependent swelling, PS exposure and protein cleavage), we could obtain detailed insight into the changes in post-translational protein modifications after stimulation of the platelets with strong Ca^2+^-mobilizing agonists; and moreover, into the suspected role of the Ca^2+^-dependent ion channel anoctamin-6 into these changes. It is relevant to note that the observed phenotypic changes in patient blood samples, which were also used for the proteomic analysis, correspond well to those earlier described for platelets from Scott patients (3, 6, 11) as well as for platelets from anoctamin-6 deficient mice (12). Another note is that the availability of platelets of only one Scott patient is a limitation of our study.

For all proteomics datasets, we systematically compared the changes induced by strong Ca^2+^-mobilizing agonists (thrombin/convulxin or ionomycin) and the differences between control and Scott platelets. Data were always compared with platelet samples from different healthy donors, in order to establish normal variability. For detecting differences in protein abundance of post-translational modification, we used relatively low threshold levels of 2 x S.D., taken from normal distribution curves, in order not to lose relevant information. On the other hand, to compensate for false-positive results, we systematically used two or three ways of data analysis to pinpoint the most consistent alterations in the proteome of Scott platelets.

Analysis of the quantitative proteome indicated that anoctamin-6 was essentially absent in Scott platelets, whereas several other (Ca^2+^ signaling) proteins were partly reduced in abundance. These changes were confirmed by PRM. Of interest is the appearance of the channel protein aquaporin-1 in Scott but not in control platelets (3 individuals). It is possible that the increased presence of aquaporin-1 is compensatory for the anoctamin-6 ion channel deficiency. This is in agreement with the recently established role of aquaporin water channels in platelet morphological changes such as ballooning (35). The link of the latter finding to the Scott syndrome needs to be confirmed, but waits for diagnosis of new patients, given that the presently included patient is the only one accessible for blood donations worldwide. Analysis of platelets from anoctamin-6 deficient mice might be an alternative (12), but will raise questions because of the different protein profile of mouse platelets in comparison to human platelets, particularly regarding activation pathways.

Quantification of 1566 phosphopeptides revealed a high similarity between Scott and control platelets after 30 min of thrombin activation. After stimulation of either type of platelets with thrombin/convulxin or ionomycin—both conditions that induce prolonged Ca^2+^ increases—we noted an overall increase in up-regulated phosphorylation sites. The possible explanation is calpain-mediated inactivation of prominent Ca^2+^-dependent phosphatases in platelets. Furthermore, with these agonists we determined on average higher phosphorylation levels in the Scott platelets compared with control platelets (see Table I). In particular, phosphorylation in the patient platelets was increased in multiple proteins regulating the cytoskeleton and implicated in phosphatidylserine exposure. Detailed analysis indicated that the majority of phosphopeptides up-regulated in stimulated control platelets was also up-regulated in the patient platelets, yet at a higher extent. Likely, this is a consequence of the longer viability of the Scott platelets after stimulation with convulxin/thrombin or ionomycin, as these cells do not swell and their membranes are less dysfunctional as a consequence of anoctamin-6 induced phospholipid scrambling. Whether aquaporin-1 can have a (compensating) role in the altered signaling is unknown. Assignment tabling indicated that mostly proteins were increased in phosphorylation that were cytoskeleton-linked or were involved in signaling (membrane receptors and channels; protein kinases and phosphatases; signaling and adaptor proteins; small GTPases and regulators). Interestingly, platelet stimulation for 30 min stimulation with thrombin alone did not result in significant phosphorylation changes, despite the fact that this agonist evoked granule secretion and long-term integrin activation. Accordingly, after long-term stimulation with thrombin, the majority of protein phosphorylation changes must have been re-equilibrated to the level at basal conditions.

ChaFRADIC analysis gave in total 1596 N-terminal peptides in patient and control platelets, 180 of which were confirmed to be calpain-regulated (corresponding to 106 proteins). We further identified a distinct set of 23 N termini (23 proteins) as caspase-regulated. Strikingly, in Scott platelets stimulated with convulxin/thrombin, the calpain-produced N termini were significantly downregulated, in particular from cytoskeleton-linked and signaling proteins. This is in agreement with Western blot analyses showing a reduced cleavage of Src kinase and talin-1 in convulxin/thrombin stimulated Scott platelets (11). It is tempting to relate the decreased protein cleavage to the increased protein phosphorylation, but this is still unclear. On the other hand, ionomycin stimulation with prolonged high cytosolic Ca^2+^ appeared to evoke more calpain-mediated protein cleavage in Scott platelets, not unlikely because of their prolonged survival allowing the Ca^2+^-dependent calpain more time for proteolytic activity. Taken together, our multipronged proteomic profiling has provided novel insight into the altered protein composition and post-translational protein machinery, which can explain major Ca^2+^- and cytoskeleton-dependent membrane alterations in Scott syndrome platelets.

## Supplementary Material

Supplemental Data

## Footnotes

^1^ The abbreviations used are:

PS: phosphatidylserine
ACD: acid-citrate glucose solution
AGC: automatic gain control
AF: Alexa Fluor
ChaFRADIC: Charge-based fractional diagonal chromatography
FDR: false discovery rate
FITC: Fluorescein isothiocyanate
PPACK: D-Phenylalanyl-prolyl-arginyl chloromethyl ketone
PRM: parallel reaction monitoring
PRP: platelet-rich plasma
PSMs: Peptide spectrum matches
SDS: sodium dodecyl sulphate
TEAB: triethylammonium bicarbonate.
