# Supplementary material for: Combined Quantification of the Global Proteome, Phosphoproteome, and Proteolytic Cleavage to Characterize Altered Platelet Functions in the Human Scott Syndrome
Source: Mol Cell Proteomics. 2016 Aug 17;15(10):3154–69. doi: 10.1074/mcp.M116.060368 (PMC5054341; doi:10.1074/mcp.M116.060368)
Supplement: Supplemental Data [file supp_15_10_3154__index.html]

Combined quantification of the global proteome, phosphoproteome and proteolytic cleavage to characterize altered platelet functions in the human Scott syndrome — Combined Quantification of the Global Proteome, Phosphoproteome, and Proteolytic Cleavage to Characterize Altered Platelet Functions in the Human Scott Syndrome — Quantitative (phospho)Proteome and N-terminome of Scott Platelets — Supplemental Data 

# Combined Quantification of the Global Proteome, Phosphoproteome, and Proteolytic Cleavage to Characterize Altered Platelet Functions in the Human Scott Syndrome

## Supplemental Data

- Supplemental Table 1 \_ Revised (.xlsx, 2.3 MB) - Supplemental Table 1 \_ Revised
- Supplemental Table 2 - phosphoproteomics (.xlsx, 994 KB) - Supplemental Table 2 - phosphoproteomics
- Supplemental Table 3 - N-terminome (.xlsx, 1.8 MB) - Supplemental Table 3 - N-terminome
- Supplemental Table 4 - PSM\_data (.xlsx, 8.8 MB) - Supplemental Table 4 - PSM\_data
- Supplemental Materials and Methods (.pdf, 110 KB) - Supplemental Materials and Methods
