# Supplementary material for: Combined Quantification of the Global Proteome, Phosphoproteome, and Proteolytic Cleavage to Characterize Altered Platelet Functions in the Human Scott Syndrome
Source: Mol Cell Proteomics. 2016 Aug 17;15(10):3154–69. doi: 10.1074/mcp.M116.060368 (PMC5054341; doi:10.1074/mcp.M116.060368)
Supplement: Supplemental Data [file 10.1074_M116.060368_mcp.M116.060368-5.pdf]

Supplemental Materials and Methods

Combined assessment of global proteome, phosphoproteome and N-terminal protein cleavage to characterize altered platelet functions in human Scott syndrome

Fiorella A. Solari[1]*, Nadine J.A. Mattheij[2]*, Julia M. Burkhart[1], Frauke Swieringa[1], Peter W. Collins[3], Judith M.E.M Cosemans[2], Albert Sickmann[1], Johan W.M. Heemskerk[2]*, René P. Zahedi[1]*

Platelet global proteome analysis using iTRAQ labels

Global proteome data were interpreted as follows. Raw data were processed with the program Proteome Discoverer 1.4 (Thermo-Fisher Scientific); data were searched against the Uniprot human database (August 2012; 20,232 target sequences). Mascot and Sequest were used as search algorithms with the following settings: *(i)* trypsin as enzyme allowing two missed cleavages, *(ii)* iTRAQ 8-plex at N-termini and lysines and carbamidomethylation of Cys residues as fixed modifications, *(iii)* oxidation of methionine as variable modification, *(iv)* mass tolerances for MS and MS/MS were set to 10 ppm and 0.02 Da, respectively. False discovery rate (FDR) estimation on the level of peptide spectrum matches was performed using the peptide validator node with filtering for 1% FDR (high confidence filter). The reporter ion quantifier node was used for iTRAQ reporter quantification. Unique proteins quantified with at least 2 unique peptides were considered.

As Proteome Discoverer only provided 7 ratios for the 8 samples, an artificial 113/113 ratio was created and set to 1.0 per protein. For each channel a median over all protein log2-ratios was calculated ($MD1_{113-121}$). Next, the median of all eight MD1 values was determined (MD2) to define normalization values (NV) per iTRAQ channel by subtracting individual MD1 values from MD2. These NV were used to compensate for individual systematic errors (i.e. unequal sample amounts derived from pipetting errors or inaccurate BCA results) and to obtain normalized ratios (NR) per protein. Next, for each protein the individual NRs were divided by

the median over all eight NRs ($MD3 = NR/MD_{(NR113-121)}$) to obtain scaled normalized abundance values (NAV) for all proteins and channels. Using the NAVs for each condition (e.g. unstimulated) ratios were determined between Scott and healthy platelets and log2-transformed. Then, for each protein the average log2 ratio and the standard deviation (SD) between Scott and healthy platelets were determined. The global median (gMD) and global SD (gSD) were determined using all proteins and were: gMD= 0.0, gSD=0.2 (log2 values).
